# Identification of Major Planktonic Sulfur Oxidizers in Stratified Freshwater Lake

**DOI:** 10.1371/journal.pone.0093877

**Published:** 2014-04-02

**Authors:** Hisaya Kojima, Tomohiro Watanabe, Tomoya Iwata, Manabu Fukui

**Affiliations:** 1 The Institute of Low Temperature Science, Hokkaido University, Sapporo, Japan; 2 Department of Environmental Sciences, University of Yamanashi, Kofu, Japan; Naval Research Laboratory, United States of America

## Abstract

Planktonic sulfur oxidizers are important constituents of ecosystems in stratified water bodies, and contribute to sulfide detoxification. In contrast to marine environments, taxonomic identities of major planktonic sulfur oxidizers in freshwater lakes still remain largely unknown. Bacterioplankton community structure was analyzed in a stratified freshwater lake, Lake Mizugaki in Japan. In the clone libraries of 16S rRNA gene, clones very closely related to a sulfur oxidizer isolated from this lake, *Sulfuritalea hydrogenivorans*, were detected in deep anoxic water, and occupied up to 12.5% in each library of different water depth. Assemblages of planktonic sulfur oxidizers were specifically analyzed by constructing clone libraries of genes involved in sulfur oxidation, *aprA*, *dsrA*, *soxB* and *sqr*. In the libraries, clones related to betaproteobacteria were detected with high frequencies, including the close relatives of *Sulfuritalea hydrogenivorans*.

## Introduction

As one of major effects of global warming on aquatic environments, enhanced stratification of water columns causes various secondary effects on ecosystems [1]–[3]. In the developed anoxic zones, activities of anaerobic microorganisms generate reduced substances which further affect biotic and abiotic environments. One of these reduced substances, sulfide originated from sulfate reduction has specifically high toxicity and can causes serious damage to various organisms. In general, sulfide in anoxic water is consumed before reaching the oxic upper water by microbes living in the boundary layer (chemocline) and lower zones. Phototrophic and chemolithotrophic sulfur oxidizers are responsible for this process, and their relative contributions greatly differ depending on availability of light.

As to sulfide detoxification by chemolithotrophs in stratified marine environments, several key sulfur oxidizers were recently identified [4]–[6]. They all belong to the phylum *Proteobacteria*, and are classified as either gammaproteobacteria or epsilonproteobacteria. On the other hand, their counterparts in freshwater ecosystems are hardly known. Although general freshwater environments are characterized by lower concentrations of sulfate and sulfide in comparison to marine environments, understanding sulfur cycles in freshwater has great importance from the aspect of water resource management since quality of available freshwater has a direct influence on human activities.

In the only previous study specifically targeting the whole community structure of freshwater planktonic sulfur oxidizers in stratified water column was performed in Lake Pavin in France, by analyzing the *aprA* gene [7]. This gene encoding adenosine-5′-phosphosulfate reductase is widely used as molecular marker to investigate sulfate reducers and sulfur oxidizers in environmental samples [8]. Among the clones of *aprA* gene belonging to lineage of sulfur oxidizers, close relatives of *Thiodictyon* sp. strain F4 were consistently detected throughout the anoxic zones of water column [7]. Indeed, all clones of sulfur oxidizers detected in lake water were closely related to this phototrophic gammaproteobacterium. In the clone libraries of 16S rRNA gene constructed from the same water samples however, sequences corresponding to *Thiodictyon* species were not detected [7]. As shown by BLAST search, these *aprA* clones are more closely related to another betaproteobacterium, *Sulfuritalea hydrogenivorans* sk43H^T^ in fact. This strain was isolated from another stratified freshwater lake (dam reservoir), Lake Mizugaki in Japan, and can grow autotrophically on thiosulfate and elemental sulfur under nitrate-reducing conditions [9]. It belongs to the family *Rhodocyclaceae* in the order *Rhodocyclales*, and its close relatives were accordingly detected in the clone libraries of 16S rRNA gene of Lake Pavin.

Although the *aprA* gene has been used as maker to analyze community structure of sulfur-oxidizing bacteria, it is not universally conserved among sulfur oxidizers. To detect diverse sulfur oxidizers with different pathways for sulfur oxidation, several marker genes have been applied to analyze environmental samples. These include *dsrA* gene encoding dissimilatory sulfite reductase, *soxB* gene encoding sulfate thioesterase/thiohydrolase, and *sqr* gene encoding sulfide:quinone oxidoreductase [10]–[12].

In the present study, assemblages of planktonic sulfur oxidizers in Lake Mizugaki were investigated by constructing clone libraries of 16S rRNA gene and above-mentioned 4 genes, *aprA*, *dsrA*, *soxB*, and *sqr*. The results obtained were compared sequences in the public database to characterize composition of sulfur oxidizers assemblages in pelagic freshwater ecosystems, which turned out to be distinct from those in marine environments.

## Materials and Methods

Lake Mizugaki is located in Yamanashi Prefecture, Japan, and stratified through the whole year [13], [14]. Measurement of environmental factors and water sample collection were performed in October 2006 as described previously [13]. Permission for field sampling was given by the Daimon and Shiokawa Dams Management Office, Yamanashi Prefectural Government. There was no any activity involving the endangered or protected species in this study. The vertical profile of irradiance (400–700 nm photosynthetically active radiation, PAR) was measured on the day of sampling at the same site (35°51.5′N, 138°30.0′E), with an underwater spherical quantum sensor LI-193 (LI-COR, Lincoln, NE, USA) to assess light availability to phototrophs.

DNA was extracted from each water sample of different depths, in the previous study [13]. From the DNA samples of 4 selected depths (5, 25, 35, and 43 m), fragments of 16S rRNA genes were amplified using universal bacterial primer pair, 27F and 1492R. PCR amplifications initiated with 2 min of denaturation at 94°C. Each thermal cycle consisted of 30 s of denaturation at 94°C, 30 s of annealing at 55°C, and 90 s of elongation at 72°C. Total cycle number was 25, and additional extension was carried out for 10 min at 72°C. The amplified fragments were ligated into the pCR2.1-TOPO vector (Invitrogen, Carlsbad, CA, USA), and the resulting vectors were transformed into competent TOP10 cells (Invitrogen). From the established libraries, clones were randomly selected and the cloned inserts were fully sequenced. Putative chimeras and other anomalous sequences were screened out by Mallard software [15], and excluded from further analysis. All clones were subjected to RDP classifier to infer their taxonomic affiliations, and grouped into operational taxonomic units (OTUs) using Mothur software [16] at a cutoff value of 0.02. Clone libraries were also constructed from PCR-amplified fragments of 4 genes involved in sulfur oxidation, *aprA*, *dsrA*, *soxB*, and *sqr*. For these genes, samples of 3 depths (25, 35, and 43 m) were analyzed. The primer pair used to amplify *aprA* gene was AprA-1-FW and AprA-5-RV, designed to detect both sulfur oxidizers and sulfate reducers [17]. PCR amplification was initiated with 3 min of denaturation at 94°C, followed by 35 cycles of 45 s at 94°C, 45 s at 55°C, and 45 s at 72°C. The final extension was carried out for 7 min at 72°C. Fragments of *dsrA*, *soxB*, and *sqr* genes were amplified with the primer pairs dsrA 625F/877R, soxB 704F/1199R, and sqr 473F/982R, respectively [18]. The PCR steps for these primer pairs were as follows: initial denaturation for 3 min at 94°C; 35 cycles of 30 s at 94°C, 30 s at 55°C, 45 s at 72°C, and a final extension for 7 min at 72°C. Cloning and sequencing were performed as described above, and the resulting sequences were translated to amino acid sequences. The amino acid sequences were aligned and then distance matrices were calculated with Poisson model using MEGA5 [19]. The calculated distance matrices were imported into Mothur to group the clones into OTUs with a cutoff value of 0.03. Phylogenetic trees were constructed with amino acid sequences of the clones representing respective OTUs, and reference sequences from the public database. Trees were constructed with the minimum-evolution method, and robustness of the trees was evaluated by bootstrap resampling analysis of 500 replicates.

The nucleotide sequences determined in this study have been assigned the DDBJ/EMBL/GenBank accession numbers AB753865-AB754337, and AB898344- AB898655.

## Results

**Figure 1:**
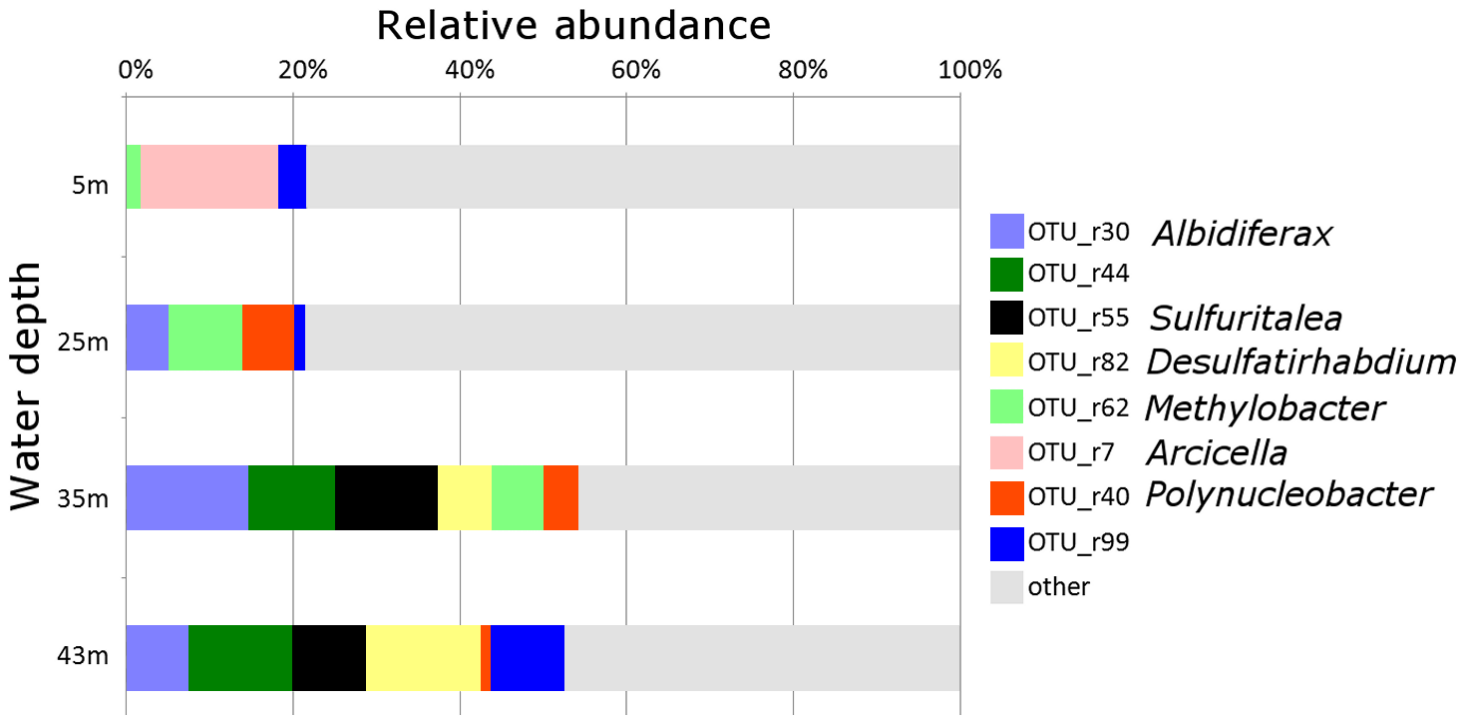
Distribution of major OTUs in the 16S rRNA gene clone libraries of different water depths. Taxonomic affiliations (genera) of OTUs were determined with RDP classifier.

As described previously [13], a clear stratification was observed at the sampling site when the water samples were obtained. In the water, PAR was attenuated exponentially and it dipped to less than 0.01% of the surface at a depth of 23 m. From the libraries of 16S rRNA gene constructed with the general primers, 321 clones were sequenced in total. There were 6 clones with anomalous sequences (5 in library of 5 m and the rest one in 25 m). By excluding these, 315 clones were grouped into 123 OTUs (Table S1). Out of these, 77 OTUs were represented by only one clone each. Depth-related distributions of 8 major OTUs (10 or more clones in total) are shown in Fig. 1. Among these, 6 OTUs could be identified at genus level (see Table S1 for details) and clones of OTU_r55 were identified as members of the genus *Sulfuritalea*. These clones were very closely related to the *Sulfuritalea hydrogenivorans* sk43H^T^, with sequence identities greater than 98%. This OTU was specifically detected in deep water, and occupied 12.5% in the clone library of 35 m (Table 1). The other major OTUs were not closely related to any known sulfur oxidizers (Fig. 1, Table S1).

**Table 1:** Compositions of the 14 clone libraries constructed in this study.

Depth	No. of *Sulfuritale hydrogenivorans*-like clone^a^/betaproteobacterial clone/total clone sequenced				
	16S rRNA gene	*aprA*	*dsrA*	*soxB*	*sqr*
5 m	0/10/60				
25 m	0/25/79	7/–b/54	2/42/42	–c	–c
35 m	12/54/96	8/–b/54	7/44/44	0/18/18	0/47/47
43 m	7/33/80	0/–b/44	13/36/51	2/49/49	0/61/61

a Clones with sequence identities >97% (16S, nucleotide; the others, amino acid), to *Sulfuritale hydrogenivorans*.

b Betaproteobacteria cannot be discriminate from gammaproteobacteria on the basis of *aprA* gene phylogeny.

c No PCR products were obtained.

**Figure 2:**
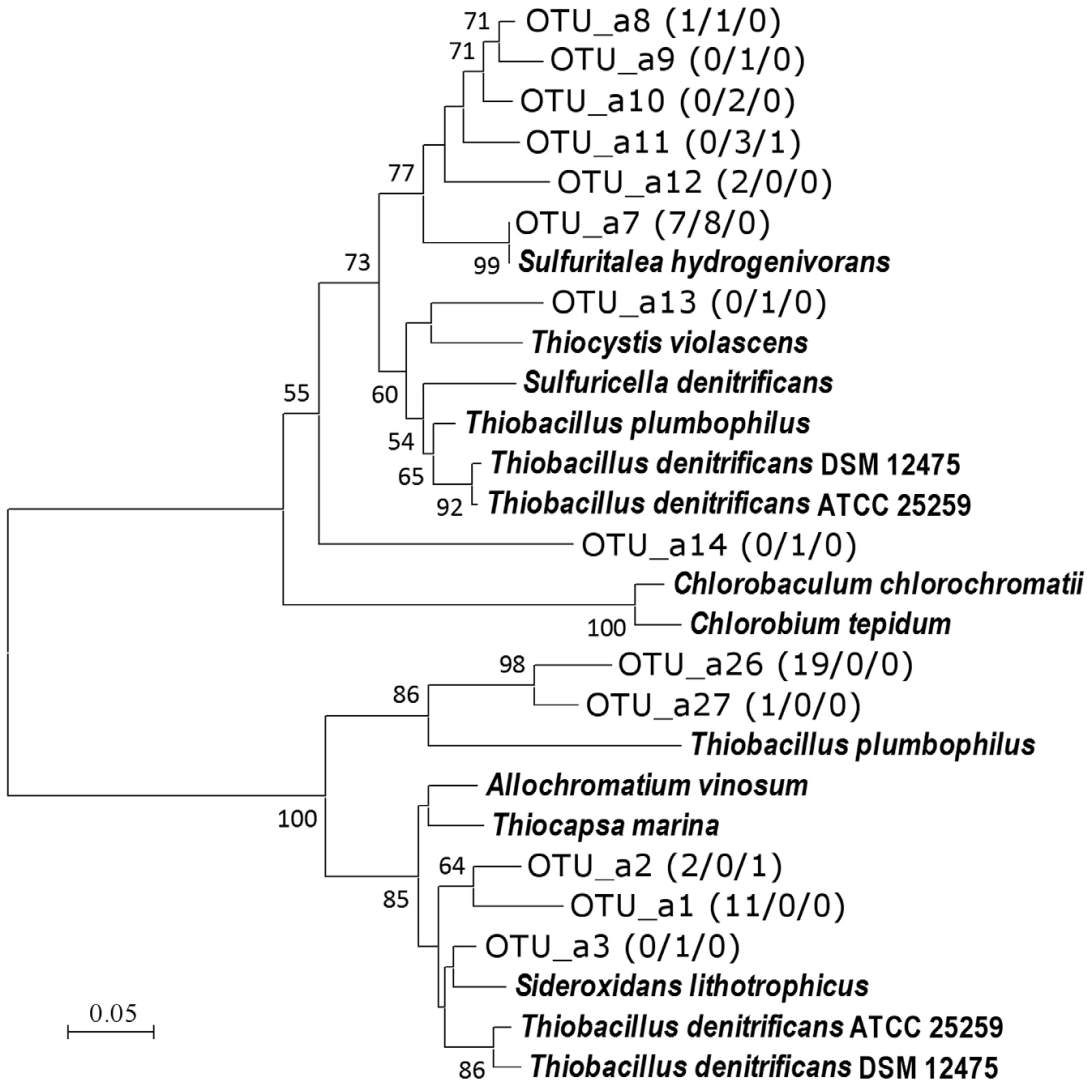
Phylogenetic relationship of AprA OTUs of sulfur oxidizers obtained in this study. Boot strap values above 50% are shown. The numbers in parentheses are the number of clones belonging to each OTU in the libraries of 25 m (n = 54), 35 m (n = 54), and 43 m (n = 44) respectively.

In the *aprA* gene analysis with the primer pair designed for concomitant detection of sulfur oxidizers and sulfate reducers, 152 clones were sequenced grouped into 27 OTUs (Table 1). Phylogenetic analysis suggested 13 OTUs were corresponding to sulfur oxidizers, and they all fell within clusters comprising betaproteobacteria and gammaproteobacteria (Fig. 2). Majority of the other clones of 14 OTUs were sulfate reducers, but functions of some OTUs were left uncertain since they had no close relatives (Table S2). From the boundary layer (25 m), relative abundance of the sulfur oxidizers decreased with increasing depth, from *ca*. 80% to 5% of the clones in each library. As shown in previous studies, it is difficult to differentiate beta- and gammaproteobacteria only by *aprA* gene sequences [8], [20]. However, one of the OTUs of sulfur oxidizers, OTU_a7 was very closely related to the *Sulfuritalea hydrogenivorans* sk43H^T^, with amino acids sequence identities of 97–100% (Fig. 2). It was the most abundant OTU among sulfur oxidizers at 35 m, but not detected in the library of 43 m dominated by relatives of sulfate reducers (Fig. 2, Table S2).

**Figure 3:**
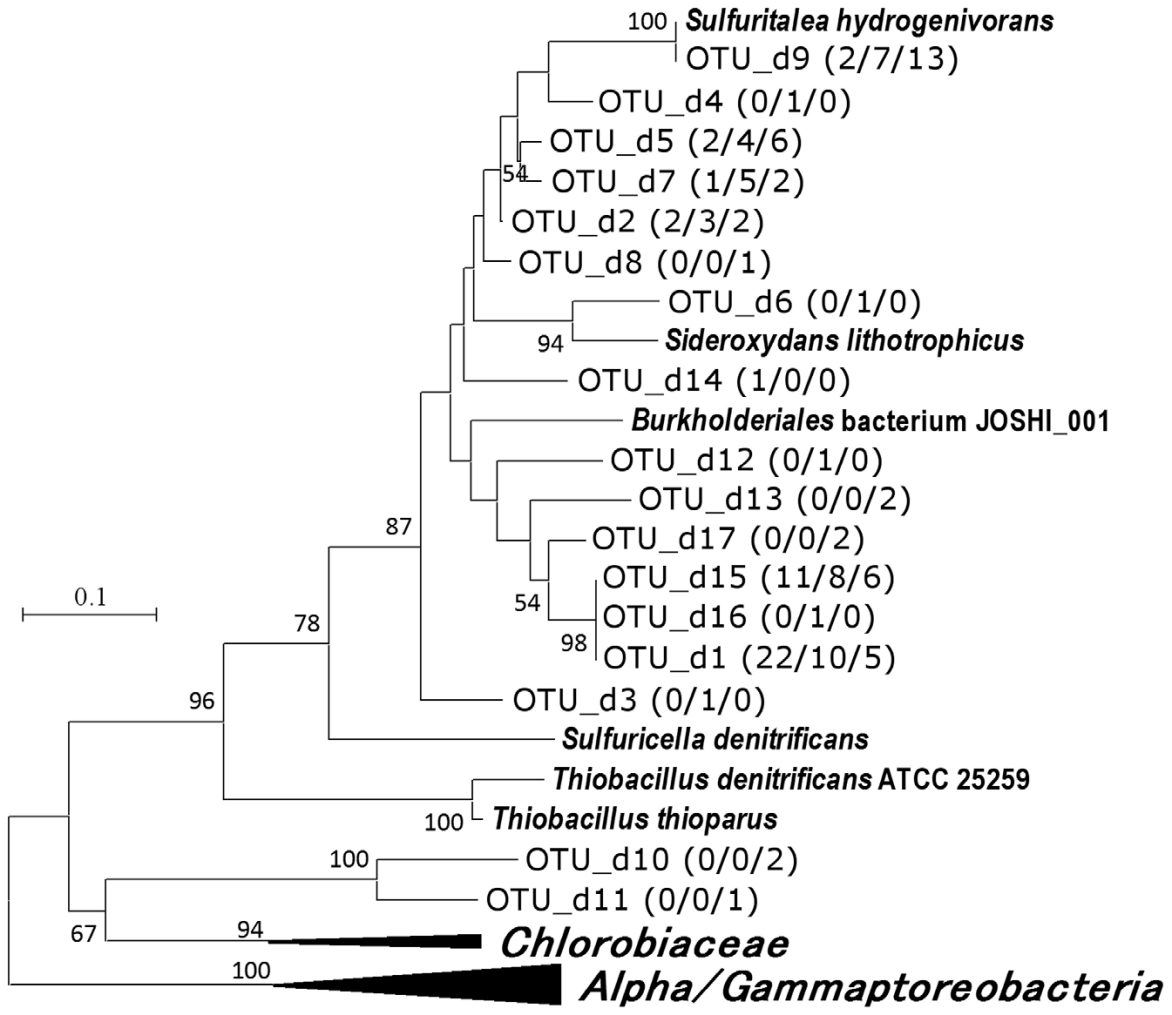
Phylogenetic relationship of DsrA OTUs of sulfur oxidizers obtained in this study. Boot strap values above 50% are shown. The numbers in parentheses are the number of clones belonging to each OTU in the libraries of 25 m (n = 42), 35 m (n = 44), and 43 m (n = 51) respectively. All entries included in the analysis to construct tree are shown in Fig. S1.

In the analysis of *dsrA* gene, 137 clones were sequenced and grouped into 22 OTUs. Although the used primer pair was designed for specific detection of sulfur oxidizers, sulfate reducers were also detected from the sample of 43 m (Table S2). The majority of the other clones (122 clones of 15 OTUs) belonged to a specific lineage including several isolated betaproteobacteria (Fig. 3). The affiliation of these OTUs to betaproteobacteria was also suggested from presence of 17-amino acid insertion, specifically observed in betaproteobacteria (Fig. S1). Only a limited number of reference sequences were available for this lineage, and majority of OTUs were only distantly related to isolated organisms. The sole exception was OTU_d9, sharing 98–100% of amino acids with *Sulfuritalea hydrogenivorans* sk43H^T^ (Fig. 3).

**Figure 4:**
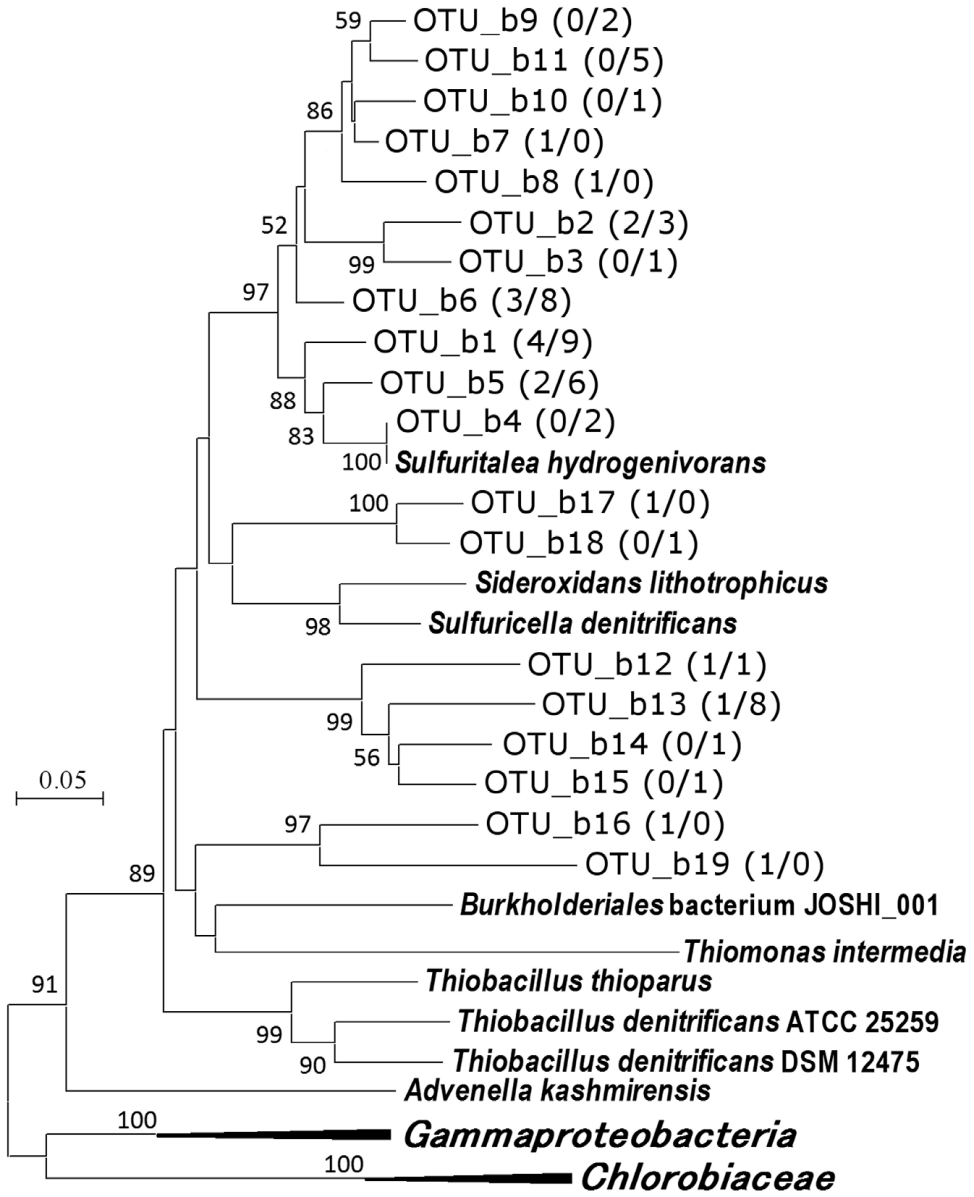
Phylogenetic relationship of SoxB OTUs obtained in this study. Boot strap values above 50% are shown. The numbers in parentheses are the number of clones belonging to each OTU in the libraries of 25 m (n = 18) and 35 m (n = 49), respectively. “*Gammaproteobacteria*” and “*Chlorobiaceae*” indicate the following species included in the analysis to construct the tree, *Allochromatium vinosum*, *Thiocystis violascens*, *Thiocapsa marina*, *Chlorobaculum chlorochromatii*, *Chlorobium tepidum*, and *Chlorobaculum parvum*.

**Figure 5:**
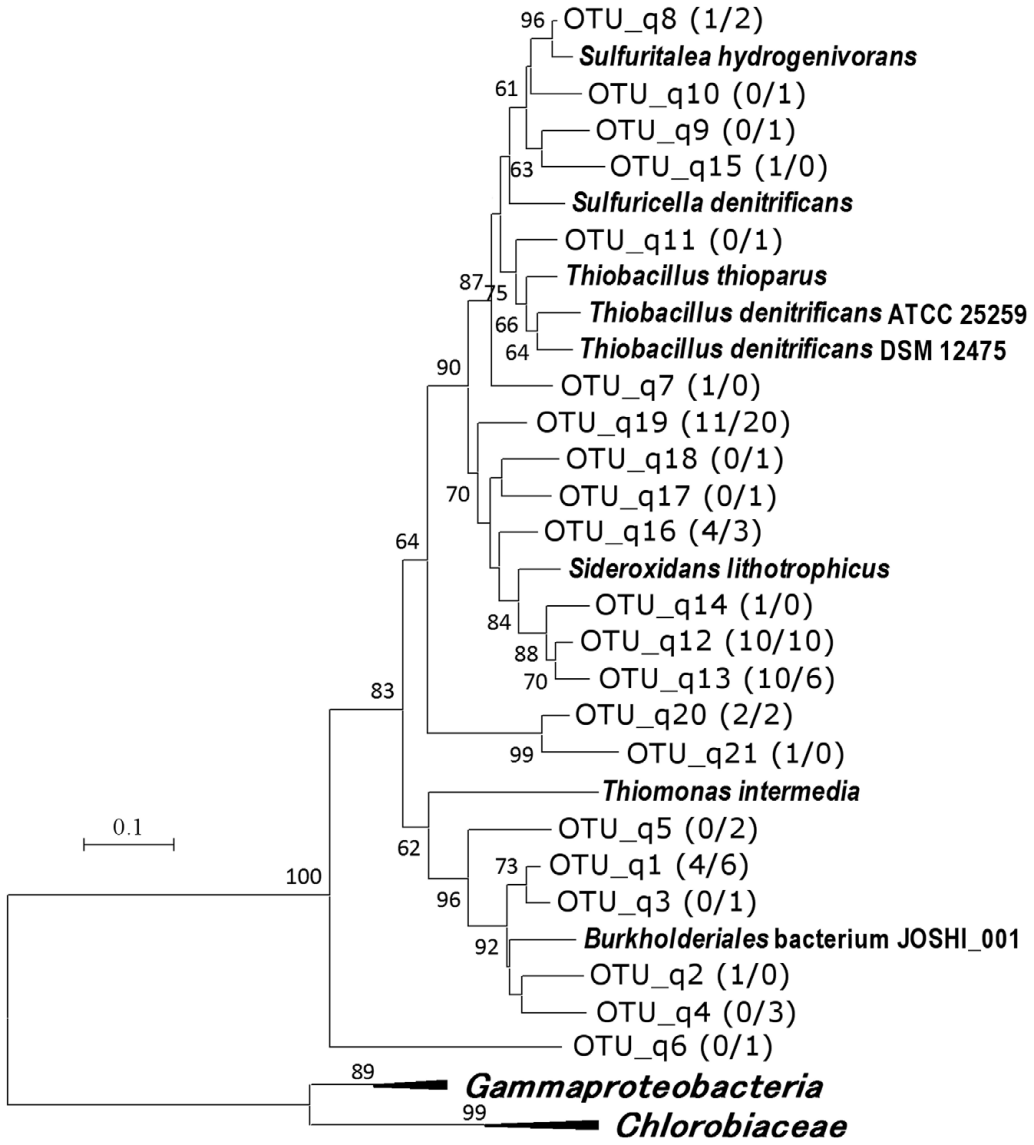
Phylogenetic relationship of Sqr OTUs obtained in this study. Boot strap values above 50% are shown. The numbers in parentheses are the number of clones belonging to each OTU in the libraries of 35 m (n = 47) and 43 m (n = 61), respectively. “*Gammaproteobacteria*” and “*Chlorobiaceae*” indicate the following species included in the analysis to construct the tree, *Allochromatium vinosum*, *Thiocystis violascens*, *Thiocapsa marina*, *Chlorobaculum chlorochromatii*, *Chlorobium tepidum*, and *Chlorobaculum parvum*.

With the primer pairs for *soxB* and *sqr* genes, no PCR products were obtained from the sample of 25 m depth. Therefore, two libraries each (35 m and 43 m) were analyzed for these genes. Phylogenetic analysis suggested all sequences obtained from these libraries were originated from betaproteobacteria (Table1, Fig. 4, Fig. 5). Clones corresponding to *Sulfuritalea hydrogenivorans* (99–100% identities) were detected in the *soxB* gene libraries of 43 m (Fig. 4).

## Discussion

The results of 16S rRNA cloning analysis targeting all bacteria suggested that *Sulfuritalea hydrogenivorans* (OTU_r55) was one of major planktonic bacteria in this lake, and its relative abundance in the bacterial community was greater in anoxic zones (Table 1, Fig. 1). There was another minor OTU of the genus *Sulfuritalea*, and it was detected in 35 m and 43 m (Table S1). As revealed by BLAST search, clones closely related to *Sulfuritalea hydrogenivorans* (>97% identities) have been detected in anoxic freshwater environments including hypolimnion of a stratified lake [7] and denitrifying enrichment culture [21]. These results suggest that *Sulfuritalea* species tend to be abundant in anoxic environments. *Sulfuritalea*-like 16S rRNA gene sequences were also detected in various environments such as nitrifying–denitrifying activated sludge [22], coal tar waste-contaminated groundwater [23], and lake sediment [24].

Although *in situ* concentration of sulfide was not determined in the present study, sulfide production in deep water was suggested from nitrate depletion shown in the previous study [13] and occurrence of sulfate reducers as indicated by analyses of *aprA* and 16S rRNA genes (Fig. 1, Table S1, Table S2). The generated sulfide and other sulfur species result from partial oxidation of sulfide may feed sulfur oxidizers including *Sulfuritalea*. In the analyses of genes involved in sulfur oxidation, clones closely related to *Sulfuritalea hydrogenivorans* sk43H^T^ were detected in samples of deep water, as OTU_a7, OTU_d9, and OTU_b4 (Table 1, Fig. 2–Fig. 4). It is likely that these clones of functional genes were originated from organisms corresponding to OTU_r55, and these organisms were sulfur oxidizers indeed.

The analyses of the functional genes also indicated that there were various sulfur oxidizers still unknown (Fig. 2–Fig. 5). These OTUs mainly belonged to lineages of betaproteobacteria, but corresponding organisms could not be identified in the clone libraries of 16S rRNA gene. They may also be important in sulfur oxidation, but there is no way to deduce their ecophysiological characteristics at present. Besides limited universality of primers, it also should be pointed out that some sulfur oxidizers lack both *aprA* and *dsrA* genes. For instance, *Sulfurimonas* species, regarded as important planktonic sulfide oxidizers [4], [7], are known to lack these genes. In the present study, two OTUs corresponding to *Sulfurimonas* species were detected in the clone library of 16S rRNA gene but they were minor fraction in comparison to *Sulfuritalea* (Table S1).

The primer pair of the *aprA* gene used in this study has been tested with numbers of reference strains in the original article [17]. These experiments demonstrated sufficient coverage of the primer pair, but presence of the inevitable bias was also shown with mixed-template PCR assays [17]. Such intensive evaluation has not been performed on the other used primer pairs of functional genes, although diverse sequences were derived from environmental samples by using them [18]. The characteristics of the primer pairs must have affected the compositions of the clone libraries, but there is no way to evaluate how they distorted the actual community structures at present.

On the whole, the results obtained in this study suggested importance of betaproteobacteria as major planktonic sulfur oxidizers. Although their detailed phylogenetic identities have not been fully elucidated, this is an important finding from one of the first molecular studies of planktonic sulfur-cycling prokaryotes in freshwater lakes. Among the betaproteobacteria, *Sulfuritalea hydrogenivorans* was consistently detected in analyses of 4 genes with considerable frequencies. Considering that its close relatives have also been detected in Lake Pavin as major planktonic sulfur oxidizers, this bacterium may be a key player in sulfur oxidation in stratified freshwater environments. On the basis of the results of present study, quantitative analysis should be done to reveal its abundance and distribution.

## Supporting Information

Fig. S1 **Multiple alignment of DsrA sequences showing the 17-amino acid insertion specifically observed in betaproteobacteria (shaded ones) and OTUs defined in this study.** For each OTU, sequence of one representative clone is shown.
(PDF)

Table S1 **Distribution and phylogenetic affiliation of all OTUs of 16S rRNA gene.**
(PDF)

Table S2 **List of AprA and DsrA OTUs which were not regarded as sulfur oxidizers.**
(PDF)
