# Supplementary material for: Identification of Major Planktonic Sulfur Oxidizers in Stratified Freshwater Lake
Source: PLoS One. 2014 Apr 2;9(4):e93877. doi: 10.1371/journal.pone.0093877 (PMC3973623; doi:10.1371/journal.pone.0093877)
Supplement: Fig. S1 — Multiple alignment of DsrA sequences showing the 17-amino acid insertion specifically observed in betaproteobacteria (shaded ones) and OTUs defined in this study. For each OTU, sequence of one representative clone is shown. (PDF) [file pone.0093877.s001.pdf]

	266	267	268	269	270	271	272	273	274	275	276	277	278	279	280	281	282	283	284	285	286	287	288	289	290	291	292	293
OTU d15	K	A	I	M	L	K	E	T	K	D	D	S	K	G	A	K	I	T	S	V	A	L	N	D	T	Q	S	L
OTU d16	K	A	I	M	L	K	E	T	K	D	V	S	K	G	A	K	I	T	C	V	A	I	N	D	T	Q	S	L
OTU d1	K	A	I	M	L	K	E	T	K	D	V	S	K	G	E	K	I	S	S	V	A	L	D	D	T	Q	S	L
OTU d17	K	T	I	M	L	K	E	T	K	D	V	A	K	G	P	K	I	S	S	V	A	I	N	D	T	Q	S	L
OTU d13	K	T	I	Q	L	K	E	I	K	D	V	A	S	G	P	K	I	S	S	V	Q	I	N	D	S	Q	C	L
OTU d14	K	A	I	M	L	K	E	I	K	D	V	G	T	G	A	K	I	S	S	V	A	V	N	D	T	Q	A	L
OTU d8	K	A	I	Q	I	K	E	T	K	D	V	S	K	G	E	K	I	S	S	V	A	L	N	D	T	Q	S	L
Sulfuritalea hydrogenivorans	K	A	L	Q	I	K	D	A	K	D	V	R	K	D	A	H	I	S	S	V	A	L	N	D	S	Q	C	L
OTU d9	K	A	L	Q	I	K	D	A	K	D	V	R	K	D	A	H	I	S	S	V	A	L	N	D	S	Q	C	L
OTU d5	K	A	I	Q	I	K	E	I	K	D	V	R	K	A	D	N	F	S	S	V	A	V	N	D	S	Q	A	L
OTU d7	K	A	I	Q	I	K	E	I	K	D	V	R	R	A	D	N	F	S	S	V	A	L	N	D	S	Q	A	L
OTU d2	K	A	I	Q	I	K	E	I	K	D	I	R	K	T	D	N	T	S	S	V	A	V	N	D	T	Q	A	L
OTU d4	K	A	I	Q	I	K	E	I	K	D	V	R	K	D	A	N	I	S	S	V	A	L	N	A	S	Q	A	L
OTU d3	K	A	M	Q	L	K	D	K	A	K	V	A	K	G	A	N	I	S	S	V	A	L	S	D	S	Q	A	L
OTU d12	H	A	I	Q	L	K	A	S	K	G	V	G	K	A	A	E	I	S	S	V	A	L	D	D	S	Q	S	L
OTU d6	K	T	F	Q	L	K	Q	I	K	N	L	K	S	G	P	S	V	S	S	V	A	V	N	D	T	H	A	I
Burkholderiales bacterium	K	A	I	Q	L	K	E	V	K	D	V	R	T	G	D	K	I	S	S	V	K	L	S	D	S	H	A	L
Sideroxydans lithotrophicus	K	T	F	Q	V	K	E	L	K	K	I	K	K	G	E	H	I	T	S	V	A	V	S	D	T	H	G	V
Sulfuricella denitrificans	K	A	L	S	L	K	N	T	K	D	V	S	T	V	E	G	I	S	S	V	A	I	N	D	T	Q	S	M
Thiobacillus denitrificans	K	A	I	T	L	V	A	S	D	K	F	A	P	S	E	T	V	S	A	A	N	L	G	D	G	N	T	L
Thiobacillus thioparus	K	A	I	T	L	V	A	S	D	K	F	K	A	S	E	H	V	S	A	A	N	L	G	D	G	N	T	L
Allochromatium vinosum	K	A	L	S	L	N	-	-	-	-	-	-	-	-	-	-	-	-	-	-	-	-	-	D	D	D	T	L
Thiorhodococcus drewsii	K	A	L	S	L	N	-	-	-	-	-	-	-	-	-	-	-	-	-	-	-	-	-	D	D	D	T	L
Thiobaca trueperi	Q	A	L	A	L	N	-	-	-	-	-	-	-	-	-	-	-	-	-	-	-	-	-	D	D	D	T	L
Thiocapsa marina	Q	A	L	A	L	N	-	-	-	-	-	-	-	-	-	-	-	-	-	-	-	-	-	D	D	D	T	L
Thiocystis gelatinosa	Q	A	L	A	L	N	-	-	-	-	-	-	-	-	-	-	-	-	-	-	-	-	-	D	D	N	T	L
Thiocystis violascens	Q	A	L	A	L	N	-	-	-	-	-	-	-	-	-	-	-	-	-	-	-	-	-	D	D	N	T	L
Marichromatium gracile	Q	A	L	S	L	N	-	-	-	-	-	-	-	-	-	-	-	-	-	-	-	-	-	D	D	D	T	L
Halochromatium salexigens	Q	A	L	S	L	N	-	-	-	-	-	-	-	-	-	-	-	-	-	-	-	-	-	D	D	D	T	L
Thiothrix nivea	Q	A	I	K	L	N	-	-	-	-	-	-	-	-	-	-	-	-	-	-	-	-	-	D	D	D	T	I
Alkalilimnicola ehrlichii	N	A	L	S	L	N	-	-	-	-	-	-	-	-	-	-	-	-	-	-	-	-	-	D	D	D	T	L
Magnetospirillum magneticum	Q	A	L	G	L	N	-	-	-	-	-	-	-	-	-	-	-	-	-	-	-	-	-	D	D	D	T	L
Rhodomicrobium vannielii	R	A	L	S	L	N	-	-	-	-	-	-	-	-	-	-	-	-	-	-	-	-	-	D	D	D	T	L
Chlorobaculum parvum	K	A	I	T	L	K	-	-	-	-	-	-	-	-	-	-	-	-	-	-	-	-	-	D	G	G	-	I
Prosthecochloris aestuarii	K	A	I	S	L	N	-	-	-	-	-	-	-	-	-	-	-	-	-	-	-	-	-	D	G	D	-	M
Chlorobium chlorochromatii	H	A	I	S	L	K	-	-	-	-	-	-	-	-	-	-	-	-	-	-	-	-	-	D	G	E	-	M
Pelodictyon phaeoclathratiform	K	A	I	A	L	K	-	-	-	-	-	-	-	-	-	-	-	-	-	-	-	-	-	E	G	E	-	M
OTU d10	R	A	L	S	L	A	-	-	-	-	-	-	-	-	-	-	-	-	-	-	-	-	-	D	G	M	-	M
OTU d11	R	A	L	S	L	A	-	-	-	-	-	-	-	-	-	-	-	-	-	-	-	-	-	D	G	A	-	M

Fig. S1. Multiple alignment of DsrA sequences showing the 17-amino acid insertion specifically observed in betaproteobacteria (shaded ones) and OTUs defined in this study. For each OTU, sequence of one representative clone is shown. Amino acid positions are numbered according to full-length DsrA sequence of *Sulfuricella denitrificans*.
