# Supplementary material for: Identification of Major Planktonic Sulfur Oxidizers in Stratified Freshwater Lake
Source: PLoS One. 2014 Apr 2;9(4):e93877. doi: 10.1371/journal.pone.0093877 (PMC3973623; doi:10.1371/journal.pone.0093877)
Supplement: Table S1 — Distribution and phylogenetic affiliation of all OTUs of 16S rRNA gene. (PDF) [file pone.0093877.s002.pdf]

Table S1. Distribution and phylogenetic affiliation of all OTUs of 16S rRNA gene

	Clone number in each library					Phylogenetic affiliation	
	5m (n=60)	25m (n=79)	35m (n=96)	43m (n=80)	total (n=315)	Phylum	genus
OTU_d1	2				2	*Bacteroidetes*	*Ferruginibacter*
OTU_d2				2	2	*Bacteroidetes*	*Ferruginibacter*
OTU_d3			2		2	*Bacteroidetes*	*Ferruginibacter*
OTU_d4			2		2	*Bacteroidetes*	*Ferruginibacter*
OTU_d5	2	1			3	*Bacteroidetes*	*Sediminibacterium*
OTU_d6	1				1	*Bacteroidetes*	*Terrimonas*
OTU_d7	10				10	*Bacteroidetes*	*Arcicella*
OTU_d8	1				1	*Bacteroidetes*	
OTU_d9	3				3	*Bacteroidetes*	*Flavobacterium*
OTU_d10	1				1	*Bacteroidetes*	*Flavobacterium*
OTU_d11	1				1	*Bacteroidetes*	*Flavobacterium*
OTU_d12			1	1	2	*Bacteroidetes*	*Prolixibacter*
OTU_d13				1	1	*Bacteroidetes*	*Prolixibacter*
OTU_d14		1			1	*Bacteroidetes*	*Prolixibacter*
OTU_d15				1	1	*Bacteroidetes*	
OTU_d16				1	1	*Bacteroidetes*	*Meniscus*
OTU_d17	1				1	*Bacteroidetes*	
OTU_d19		1			1	*Bacteroidetes*	
OTU_d20		1			1	*Bacteroidetes*	
OTU_d21	1				1	*Bacteroidetes*	
OTU_d22		1			1	*Bacteroidetes*	
OTU_d23		2			2	*Bacteroidetes*	
OTU_d24			1	1	2	*Bacteroidetes*	
OTU_d25		1			1	*Bacteroidetes*	
OTU_d26	3				3	*Bacteroidetes*	
OTU_d27		1			1	*Bacteroidetes*	*Fluviicola*
OTU_d28	1				1	*Bacteroidetes*	*Fluviicola*
OTU_d30		4	14	6	24	*Proteobacteria*	*Albidiferax*
OTU_d31	1				1	*Proteobacteria*	*Albidiferax*
OTU_d32	1				1	*Proteobacteria*	
OTU_d33		1			1	*Proteobacteria*	*Polaromonas*
OTU_d34	3	1			4	*Proteobacteria*	*Limnohabitans*
OTU_d36	1				1	*Proteobacteria*	
OTU_d37		1			1	*Proteobacteria*	*Limnohabitans*
OTU_d38		4			4	*Proteobacteria*	*Polynucleobacter*
OTU_d39	1				1	*Proteobacteria*	*Polynucleobacter*
OTU_d40		5	4	1	10	*Proteobacteria*	*Polynucleobacter*
OTU_d41	1				1	*Proteobacteria*	*Polynucleobacter*
OTU_d43		2			2	*Proteobacteria*	
OTU_d44			10	10	20	*Proteobacteria*	
OTU_d45			1		1	*Proteobacteria*	
OTU_d46			1		1	*Proteobacteria*	
OTU_d47				2	2	*Proteobacteria*	
OTU_d48			5		5	*Proteobacteria*	
OTU_d49		3			3	*Proteobacteria*	*Nitrosospira*
OTU_d50		1	3	3	7	*Proteobacteria*	*Methylotenera*
OTU_d51		1			1	*Proteobacteria*	
OTU_d52			2		2	*Proteobacteria*	
OTU_d53				1	1	*Proteobacteria*	*Dechloromonas*
OTU_d54		1			1	*Proteobacteria*	
OTU_d55			12	7	19	*Proteobacteria*	*Sulfuritalea*
OTU_d56			1	1	2	*Proteobacteria*	*Sulfuritalea*
OTU_d57			1		1	*Proteobacteria*	*Sterolibacterium*
OTU_d58				1	1	*Proteobacteria*	

OTU_d59				1	1	*Proteobacteria*	
OTU_d60	2				2	*Proteobacteria*	
OTU_d61		1			1	*Proteobacteria*	
OTU_d62	1	7	6		14	*Proteobacteria*	*Methylobacter*
OTU_d63		5	1	1	7	*Proteobacteria*	*Methylobacter*
OTU_d64	1	4			5	*Proteobacteria*	
OTU_d65			1		1	*Proteobacteria*	
OTU_d66				1	1	*Proteobacteria*	
OTU_d67		4	1		5	*Proteobacteria*	*Legionella*
OTU_d68		1			1	*Proteobacteria*	
OTU_d69			1		1	*Proteobacteria*	*Methylocaldum*
OTU_d70	1				1	*Proteobacteria*	*Methylobacterium*
OTU_d71				1	1	*Proteobacteria*	
OTU_d72	1				1	*Proteobacteria*	
OTU_d74	1				1	*Proteobacteria*	
OTU_d75			3		3	*Proteobacteria*	*Sulfurimonas*
OTU_d76				1	1	*Proteobacteria*	*Sulfurimonas*
OTU_d77		1			1		
OTU_d78				1	1		
OTU_d79			2	1	3	*Acidobacteria*	*Geothrix*
OTU_d80			1		1		
OTU_d81		1			1	*Proteobacteria*	
OTU_d82			6	11	17	*Proteobacteria*	*Desulfatirhabdium*
OTU_d83			1	3	4	*Proteobacteria*	*Geobacter*
OTU_d84				3	3	*Proteobacteria*	*Geobacter*
OTU_d85				1	1	*Proteobacteria*	*Geobacter*
OTU_d86				1	1	*Proteobacteria*	*Syntrophus*
OTU_d87		1			1	*Proteobacteria*	*Desulfomicrobium*
OTU_d88			3		3	*Proteobacteria*	*Desulfurivibrio*
OTU_d89			1		1	*Proteobacteria*	
OTU_d90				1	1	*Proteobacteria*	*Desulfobulbus*
OTU_d91				1	1	*Proteobacteria*	
OTU_d92	1	1			2	Chloroplast	*Cryptomonadaceae*
OTU_d93	1				1	*Cyanobacteria*	
OTU_d94	1				1	*Cyanobacteria*	GpIIa
OTU_d95	1				1	*Cyanobacteria*	GpIIa
OTU_d96	2	2			4	*Actinobacteria*	*Ilumatobacter*
OTU_d97		1			1	*Actinobacteria*	*Ilumatobacter*
OTU_d98	1				1	*Actinobacteria*	*Acidimicrobineae*
OTU_d99	2	1		7	10	*Actinobacteria*	
OTU_d100	1				1	*Actinobacteria*	
OTU_d101	1				1	*Actinobacteria*	
OTU_d102	1				1	*Actinobacteria*	
OTU_d103			3		3	*Gemmatimonade*	*Gemmatimonas*
OTU_d104		1			1		
OTU_d105	3				3	*Armatimonadete*	*Armatimonas/Armatimonadetes*_gp1
OTU_d106		2			2	*Chloroflexi*	
OTU_d107				1	1	*Chloroflexi*	
OTU_d108	1				1	*Firmicutes*	
OTU_d109				1	1	*Firmicutes*	*Acetivibrio*
OTU_d110				1	1	*Firmicutes*	*Anaerovorax*
OTU_d111		2			2	*Verrucomicrobia*	*Cerasicoccus*
OTU_d112	1				1	*Verrucomicrobia*	
OTU_d113	1				1	*Verrucomicrobia*	*Opitutus*
OTU_d114		1			1	*Verrucomicrobia*	
OTU_d115		1			1	*Verrucomicrobia*	*Verrucomicrobium*
OTU_d116			1		1	*Verrucomicrobia*	Spartobacteria_genera_incertae_sedis
OTU_d117		1			1	*Verrucomicrobia*	Subdivision3_genera_incertae_sedis
OTU_d118				1	1	*Verrucomicrobia*	Subdivision3_genera_incertae_sedis

OTU_d119		1		1		
OTU_d120		1	1	2	*Lentisphaerae*	
OTU_d121		1		1	*Lentisphaerae*	*Victivallis*
OTU_d122	3			3	*Planctomycetes*	*Planctomyces*
OTU_d123	1			1	*Planctomycetes*	*Schlesneria*
OTU_d124	1			1		
OTU_d125			1	1		
OTU_d126	1			1	*Planctomycetes*	*Phycisphaera*
OTU_d127	2			2		
OTU_d128		2		2		
