# Supplementary material for: Identification of Major Planktonic Sulfur Oxidizers in Stratified Freshwater Lake
Source: PLoS One. 2014 Apr 2;9(4):e93877. doi: 10.1371/journal.pone.0093877 (PMC3973623; doi:10.1371/journal.pone.0093877)
Supplement: Table S2 — List of AprA and DsrA OTUs which were not regarded as sulfur oxidizers. (PDF) [file pone.0093877.s003.pdf]

Table S2. List of AprA and DsrA OTUs which were not regarded as sulfur oxidizers.

	OTU name	No. of clones in each library			Closest species with validated names	% identity
		25m	35m	43m		
AprA	OTU_a4	2	0	0	*Thermodesulfovibrio islandicus*	72
	OTU_a5	0	1	0	*Thermodesulfovibrio islandicus*	77
	OTU_a6	0	0	1	*Thermodesulfovibrio islandicus*	78
	OTU_a15	0	1	0	*Desulfovibrio fructosovorans*	81
	OTU_a16	2	1	0	*Desulfobulbus rhabdoformis*	91
	OTU_a17	5	0	0	*Desulfobulbus rhabdoformis*	92-94
	OTU_a18	0	8	8	*Desulfobulbus elongatus*	92-94
	OTU_a19	0	1	0	*Desulfobulbus elongatus*	93
	OTU_a20	0	7	1	*Desulfocapsa thiozymogenes*	92-93
	OTU_a21	0	1	0	*Desulfocapsa thiozymogenes*	92
	OTU_a22	0	0	1	*Desulfocapsa thiozymogenes*	92
	OTU_a23	0	1	0	*Desulfobacterium catecholicum*	92
	OTU_a24	2	14	31	*Desulfosarcina variabilis*	90-92
	OTU_a25	0	1	0	*Desulfobacterium indolicum*	95
DsrA	OTU_d18	0	0	8	*Desulforhopalus singaporensis*	80-81
	OTU_d19	0	0	1	*Desulforhopalus singaporensis*	80
	OTU_d20	0	0	1	*Desulfurivibrio alkaliphilus*	73
	OTU_d21	0	0	1	*Desulfacinum infernum*	53
	OTU_d22	0	0	1	*Pelotomaculum propionicicum*	58
